# Early-onset diabetes with low utilization of lipid as an energy source carrying a rare missense mutation in the *CEL* gene

**DOI:** 10.1530/EDM-24-0151

**Published:** 2025-08-18

**Authors:** Ayana Fujii, Hiroko Nakabayashi, Yuko Nagao, Masaru Akiyama, Akihiko Taguchi, Kaito Yorimoto, Risako Hamada, Issei Saeki, Naoki Yamamoto, Taro Takami, Kenji Watanabe, Yoichi Mizukami, Yasuharu Ohta

**Affiliations:** ^1^ Department of Endocrinology, Metabolism, Hematological Science and Therapeutics, Yamaguchi University, Graduate School of Medicine, Ube, Japan; ^2^ Department of Gastroenterology and Hepatology, Yamaguchi University, Graduate School of Medicine, Ube, Japan; ^3^ Yamaguchi University Health Science Center, Yamaguchi, Japan; ^4^ Yamaguchi University Center for Gene Research, Ube, Japan

**Keywords:** MASLD, pancreatic exocrine function, carboxyl ester lipase, early-onset diabetes, low utilization of lipid

## Abstract

**Summary:**

Carboxyl ester lipase (*CEL*) is a major component of pancreatic juice and is responsible for the duodenal hydrolysis of cholesteryl esters. Maturity-onset diabetes of the young (MODY) is a form of diabetes mellitus characterized by early onset and dominant inheritance of beta-cell dysfunction. *CEL* gene mutations cause the type of MODY denoted as MODY8. Herein, we describe a Japanese patient who harbored a heterozygous A689P mutation in the variable number of tandem repeats (VNTRs)-containing exon 11 of the *CEL* gene. The patient was not obese and his diabetes was characterized by onset in late adolescence, impaired insulin secretion and metabolic dysfunction-associated steatotic liver disease (MASLD). The C-terminal region of *CEL* has been postulated to be critical for its secretion and activity. Therefore, the A689P mutation may cause pancreatic exocrine insufficiency and eventually contribute to MASLD, which is associated with reduced lipid catabolism. MODY8 is also considered to be a protein-misfolding disease because a heterozygous single nucleotide deletion causes the production of mutant CEL protein leading to diabetes and exocrine dysfunction. In the present case, MASLD and diabetes characterized by impaired insulin secretion were observed. The CEL A689P missense mutation will expand the known genotype–phenotype correlation in diabetes if it can be demonstrated that the variant is pathogenic.

**Learning points:**

- The *CEL* gene encodes the digestive enzyme carboxyl ester lipase, also known as bile salt-stimulated/dependent lipase.
- *CEL* is expressed in pancreatic acinar tissue but not in pancreatic β cells.
- MODY caused by mutations in the *CEL* gene (MODY8) is characterized by dominantly inherited diabetes mellitus manifesting in early adulthood.
- A classical feature of MODY8 is pancreatic exocrine dysfunction, often with onset in childhood.
- Known pathogenic mutations in the *CEL* gene affect the variable number tandem repeat (VNTR) region in exon 11.
- Our case suggests that some missense mutations of the *CEL* VNTR could have a phenotypic implication by being associated with impaired glucose-stimulated insulin secretion and reduced utilization of lipid as an energy source which leads to MASLD.

## Background

Fourteen distinct pathogenic genes associated with MODY have been identified and may account for 1–2% of the overall prevalence of diabetes (1, 2). MODY8 is caused by mutations in *CEL* and is a genetic disorder characterized by pancreatic exocrine dysfunction with childhood onset and diabetes starting in adulthood (3, 4). The last exon contains a guanine- and cytosine-rich VNTR region, comprised of 7–23 nearly identical, repeated sequences, each encoding 11-amino acids in the protein tail (5, 6). Regarding the disease mechanism of MODY8, it has been suggested that mutations in the VNTR cause abnormal aggregation of the mutant protein, which increases endoplasmic reticulum (ER) stress, leading to pancreatic exocrine dysfunction and diabetes (7, 8). In our present case, a missense mutation (A689P) in the CEL VNTR region was identified by whole-genome analysis, and MASLD with impaired insulin secretion was observed. MASLD has not been mentioned in reports of MODY8 but is assumed to be related to reduced utilization of lipids as an energy source due to pancreatic exocrine dysfunction. Herein, we describe a case of impaired glucose-stimulated insulin secretion and a rare *CEL* variant, and discuss relevant clinical findings, including MASLD.

## Case presentation

A 51-year-old Japanese man with diabetes was referred to our department because his blood glucose was too elevated for cervical spine surgery to be performed safely. The patient was admitted to our department for a short stay before surgery to achieve adequate blood glucose control. Blood glucose elevation had first been detected at age 35 years. He was initially prescribed oral medications, which had been continued for approximately 5 years. Insulin treatment was initiated at age 40 and he continues to take insulin. His mother had diabetes, but his grandmother and siblings did not, and there was no other known family history of diabetes.

**Figure 1:**
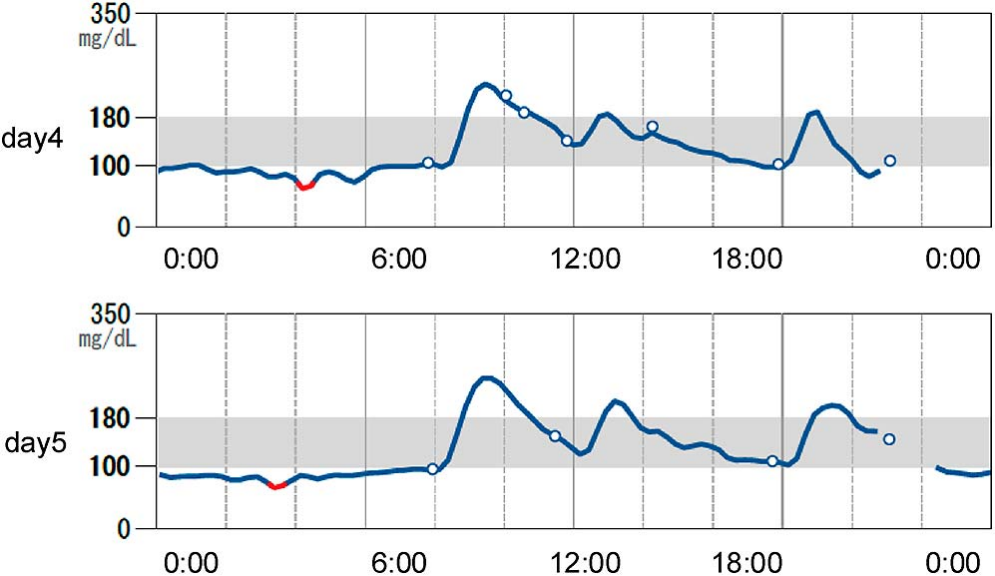
The *isCGM* results for days 4–5 of treatment with insulin. *isCGM*, intermittent continuous glucose monitoring.

On admission, his BMI was 21.6 kg/m^2^ (height: 163.3 cm, weight: 58.4 kg). No signs of diabetic retinopathy or nephropathy (7.1 mg/day of microalbuminuria) were detected. His fasting serum C-peptide and blood glucose levels were 0.21 nmol/L and 4.7 mmol/L, respectively, while his urinary excretion of C-peptide was 2.8 nmol/day, indicating modestly impaired insulin secretion (Table 1). However, the changes in C-peptide observed during the glucagon stimulation test (ΔCPR; 0.4 nmol/L) were generally normal, suggesting that glucose-dependent insulin secretion was specifically reduced in this case. Intermittent continuous glucose monitoring (isCGM) with FreeStyle Libre (Abbott Diabetes Care, USA) was performed while continuing insulin therapy, and the insulin dosage at the time was 6.0 U of aspart before each meal, while that of degludec was 6.0 U in the evening. As shown in Fig. 1, fasting blood glucose levels were generally low while postprandial glucose levels were somewhat elevated after breakfast despite administration of insulin aspart before meals, suggesting that he suffered from impaired glucose-stimulated insulin secretion and reduced hepatic glucose production.

**Table 1:** Laboratory data for this patient.

	Normal values	Results
Albumin (g/dL)	4.1–5.1	3.8
AST (U/L)	13–30	29
ALT (U/L)	10–42	67
ALP (U/L)	38–113	56
γ-GTP (U/L)	13–64	23
Total bilirubin (μmol/L)	6.8–26	17
Direct bilirubin (μmol/L)	1.7–5.1	5.1
Cholesterol (mmol/L)	3.7–6.4	3.5
Triglyceride (mmol/L)	0.5–2.6	0.8
HDL-cholesterol (mmol/L)	1.0–2.3	1.2
LDL-cholesterol (mmol/L)	1.7–4.2	1.9
Creatinin (μmol/L)	57–95	80
BUN (mmol/L)	2.9–7.1	5.4
Uric acid (μmol/L)	220–464	357
Lactic acid (mmol/L)	0.44–1.78	1.55
Pyruvate (μmol/L)	45–113	8
Amylase (U/L)	44–132	135
Pancreatic lipase (U/L)	17–50	35
Lipase (U/L)	13–55	26
Sodium (mEq/L)	138–145	141
Potassium (mEq/L)	3.6–4.8	4.4
Chloride (mEq/L)	101–108	107
Fasting plasma glucose (mmol/L)	4.1–6.1	4.7
Fasting plasma C-peptide (nmol/L)	0.17–0.66	0.21
Urine C-peptide (nmol/day)	16–33	2.8
HbA1c (%)	4.9–6.0	7.6

The patient had no complaints of abdominal pain, diarrhea or fatty stools that would suggest a clinical diagnosis of exocrine pancreatic insufficiency. Serum lipase and pancreatic amylase levels were also within normal ranges (Table 1). However, enzyme activity in duodenal juice was not assessed.

He was not obese, but alanine amino transferase (ALT) was elevated (67 U/L) and ultrasound revealed MASLD. No obvious morphological abnormalities of the pancreas were detected. On admission, the serum lactic acid (1.55 mmol/L) level was within normal range, probably because metformin had been discontinued, while serum pyruvate remained very low (8 μmol/L) and the lactic acid-to-pyruvate ratio was very high (194).

**Figure 2:**
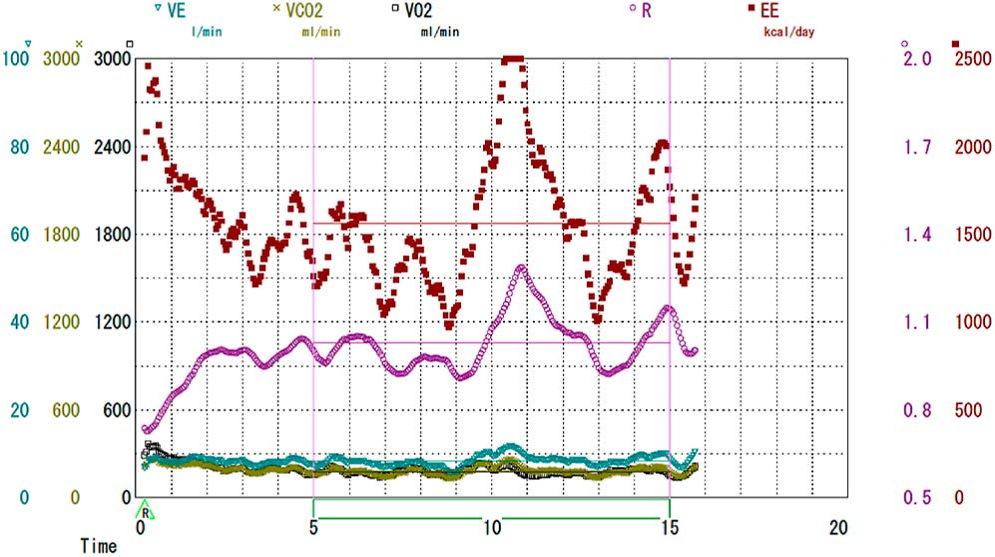
Recorded: VE, VCO_2_, VO_2_, R, and EE. EE was calculated from recorded VCO_2_ and VO_2_. VE, minute ventilation; VCO_2_, carbon dioxide production; VO_2_, oxygen consumption; R, respiratory quotient; EE, energy expenditure.

The resting minute ventilation (VE), carbon dioxide production (VCO_2_), oxygen consumption (VO_2_), respiratory quotient (R) and energy expenditure (EE) are shown in Fig. 2 and Table 2. R was very high, indicating reduced rates of lipid oxidation. Table 3 illustrates the relative participations of glucose, lipid, and protein in energy expenditure, as calculated by the formula devised by Elwyn *et al.* (1949) also known as Weir equation (9). Interestingly, energy derived from lipid utilization was extremely low (3.3%) (Table 3), which might be associated with impaired glucose-stimulated insulin secretion, MASLD and reduced gluconeogenesis.

**Table 2:** Patient characteristics based on indirect calorimetry measurements. Results are shown as averages of 10 min measurements.

	Results
VE (L/min)	8.4
VCO_2_ (mL/min)	184
VO_2_ (mL/min)	178
R	1.03
EE (kcal/day)	1,564

VE, minute ventilation; VCO_2_, carbon dioxide production; VO_2_, oxygen consumption; R, respiratory quotient; EE, energy expenditure.

**Table 3:** Percentages of carbohydrate, lipid, and protein expended as energy. Values recorded as percentage burned.

	Results
Carbohydrate	85.7
Lipid	3.3
Protein	11.1

## Investigation

**Figure 3:**
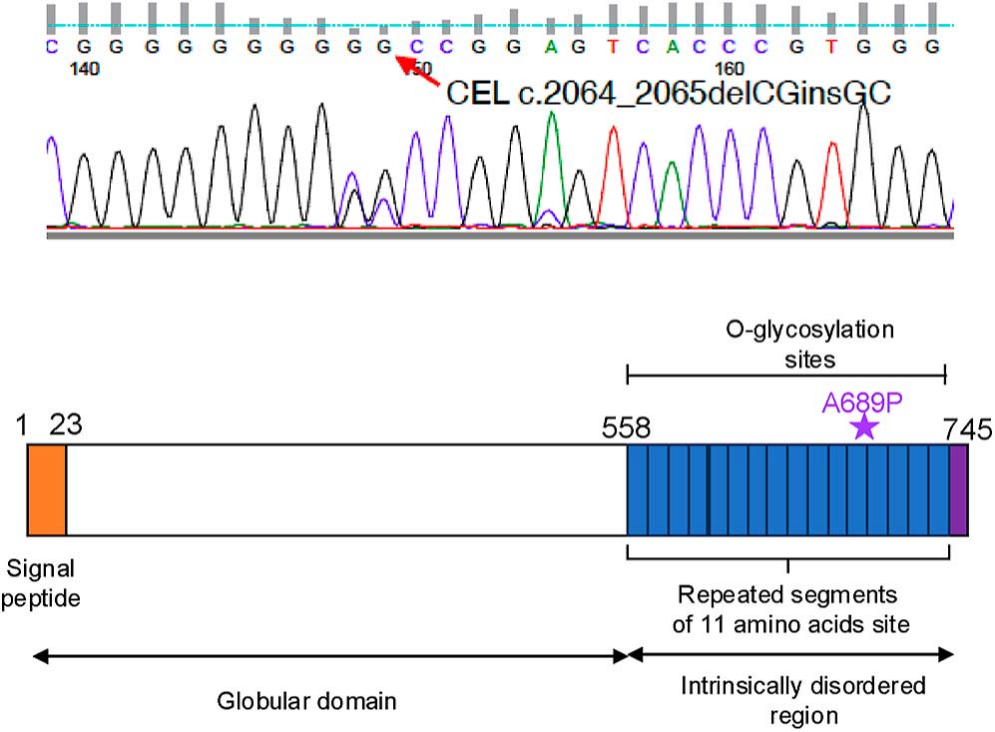
Sequence analysis of CEL from the patient. Chromatogram represents Sanger sequencing of patient’s CEL gene, confirming the heterozygous deletion and insertion mutations (upper). Schematic structure of the CEL protein with functional domain and the mutation indicated (bottom).

We sought to determine whether any mitochondrial (mt) DNA mutations were present in the blood of our patient but no pathogenic mtDNA mutation was detected. Since most mitochondrial proteins are encoded by nuclear genes, we performed a whole-genome analysis to search for genomic variations, possibly affecting glucose and lipid metabolism, including MODY relevant variants. Whole-genome libraries were constructed using the NEBNext Ultra Ⅱ DNA Library Prep Kit for Illumina (NEB) and sequenced on the NovaSeq 6000 (Illumina, CA, USA) to generate 2 × 150 bp reads. All sequencing reads were evaluated for quality using the ShortRead package. To assist with the interpretation of variant pathogenicity, we referred to the Qiagen database. To assess MODY relevant variants, we started by filtering 9,684,191 variants. We filtered out common variants to obtain 374,868 variants. We next focused on 286,119 variants predicted to be deleterious. To facilitate variant interpretation, we focused on 52,638 variants that included known MODY genes and others implicated in diabetes. Then, we again filtered out common variants, focused on 40 variants predicted to have pathogenicity and selected 31 variants that showed combined annotation-dependent depletion scores greater than 20. In addition, PolyPhen2 functional prediction revealed the c.2064_2065delCGinsGC mutation (A689P) to probably have a damaging effect on CEL protein. As shown in Fig. 3, this variant was also confirmed by Sanger sequencing and was found to be located at an intrinsically disordered region.

## Discussion

We detected a rare missense variant (c.2064_2065delCGinsGC) in the *CEL* gene. This was a substitution of an alanine with a proline in the *CEL* VNTR region. The *CEL* gene is recognized as the causative gene for MODY8. The present case did not meet the MODY8 criteria, and moreover, his diabetes was also characterized by late adolescent onset (4, 10). Due to the heterogeneity of MODY, traditional criteria might well miss most cases. It is estimated that more than 80% of MODY patients are incorrectly diagnosed as having type 1 or type 2 diabetes (11), and the present case also had never been given a diagnosis of MODY. More genetic testing, focusing on accurate diagnosis, of newly diagnosed diabetic patients ranging in age from 15 to 35 years may be needed to provide more effective treatment for MODY patients.

Many studies have suggested that communication between exocrine and endocrine cells may be critical. In fact, the incidence of diabetes secondary to exocrine dysfunction ranges from 25 to 80% (12). In the present case, insulin secretion was significantly reduced. The proposed pathogenic mechanism underlying MODY8 involves the abnormal aggregation of mutant proteins, which increases ER stress in acinar cells, subsequently leading to the onset of diabetes associated with impaired insulin secretion (7, 8).

The lactate/pyruvate ratio was significantly increased in our patient and further examinations showed that lipid utilization as an energy substrate was extremely low, at only 3.3%, indicating lipid catabolism to be severely impaired. In addition, the patient exhibited signs of MASLD while the patient was not obese. MASLD, which appears to be associated with impaired pancreatic exocrine function, is reportedly observed in approximately 30% of patients after extensive pancreatectomy (13, 14, 15). Therefore, MASLD was considered to be a consequence of decreased lipid catabolism resulting from pancreatic exocrine insufficiency in the present case.

We must acknowledge several limitations. First, we were not able analyze the enzyme activities of lipase and amylase in duodenal juice samples. Reportedly, there are no differences in baseline values of the movement of water in pancreatic head tissue, but peak levels of enzyme activity in secretin-stimulated duodenal juice were found to be markedly reduced in MODY8 patients (16). Therefore, pancreatic exocrine dysfunction could not be ruled out in this case, despite serum enzyme activities being within the normal ranges. Second, also related to the above, pancreatic enzyme replacement therapy (PERT) has not yet been attempted for the management of this patient. PERT also reportedly reduces the incidence of MASLD after pancreaticoduodenectomy (15). We can reasonably speculate that PERT would ameliorate not only MASLD but also impaired insulin secretion in association with improved exocrine function.

The present case is not MODY8, but early-onset diabetes with low utilization of lipid as an energy source, in which a rare missense mutation in the CEL gene is observed. The CEL A689P missense mutation will expand the known genotype–phenotype correlation in diabetes if it can be demonstrated that the variant is pathogenic.

## Declaration of interest

The authors declare that there is no conflict of interest that could be perceived as prejudicing the impartiality of the work reported.

## Funding

This work was supported by the Japan Society for the Promotion of Sciencehttps://doi.org/10.13039/501100001691 (Grant No. 22K084626 to YO).

## Patient consent

Written informed consent for the publication of the clinical data was obtained from the patient, and this study was approved by the Yamaguchi University Hospital Ethics Committee (Protocol No. 2021-158-2).

## Author contribution statement

AF, HN, MA, RH, HM and YO were in charge of the medical care provided to this patient. KW and YM extracted genomic DNA and conducted whole genome sequencing analyses. YK and AT performed three-dimensional structure analysis of CEL. YN, AT, IS, NY and TT contributed to medical care and manuscript preparation. AF and YO wrote the manuscript. IS, NY, TT and YM supervised this project. All authors read and approved the final manuscript.
